# Expression of Monocarboxylate Transporter 1 in Immunosuppressive Macrophages Is Associated With the Poor Prognosis in Breast Cancer

**DOI:** 10.3389/fonc.2020.574787

**Published:** 2020-10-16

**Authors:** Bei Li, Qian Yang, Zhiyu Li, Zhiliang Xu, Si Sun, Qi Wu, Shengrong Sun

**Affiliations:** ^1^Department of Pathology, Renmin Hospital of Wuhan University, Wuhan, China; ^2^Department of Breast and Thyroid Surgery, Renmin Hospital of Wuhan University, Wuhan, China; ^3^Department of Clinical Laboratory, Renmin Hospital of Wuhan University, Wuhan, China

**Keywords:** breast cancer, tumor-associated macrophage, MCT1, CD163, recurrence-free survival

## Abstract

Monocarboxylate transporter 1 (MCT1) participates in the transport of lactate to facilitate metabolic reprogramming during tumor progression. Tumor-associated macrophages (TAMs) are also involved in the inflammatory adaptation of the tumor microenvironment (TME). This study aimed to determine the correlation between metabolite changes and the polarization of macrophages in the TME. We demonstrated that the expression of CD163 on macrophages was significantly higher in breast cancer tissues than in normal tissues, especially in the HER2 subtype, although it was not statistically associated with recurrence-free survival (RFS). The presence of MCT1^+^ and CD163^+^ macrophages in the invasive margin was significantly correlated with decreased RFS. A significant correlation existed between MCT1 and CD163 expression in the margin, and high infiltration of MCT1^+^CD163^+^ macrophages into the margin predicted rapid progression and poor survival outcomes for breast cancer patients. These data suggested that MCT1 at least partially promoted the alternative polarization of macrophages to inhibit antitumor immunity, and blocking this interaction may be a promising method for breast cancer therapy.

## Introduction

The tumor microenvironment (TME) is a heterogeneous ecosystem, including infiltrating immune cells, mesenchymal support cells, and matrix components (1). With the metabolic and inflammatory reprogramming of tumor cells during cancer progression, the TME is converted into an advantageous microenvironment with altered generation of metabolites, such as lactate, pyruvate and ketone bodies, and adaptive infiltration of tumor-infiltrating lymphocytes. Macrophages are one of the important immune cells recruited to the TME, which have two subsets, “classically activated” M1 macrophages and “alternatively activated” M2 macrophages (2). Generally, M1 macrophages are thought to be proinflammatory and are characterized by high expression of proinflammatory factors, such as interleukin (IL)-12, nitric oxide synthase 2 (NOS2), and tumor necrosis factor (TNF)-α. However, M2 macrophages are considered to be immunosuppressive and generate high levels of anti-inflammatory cytokines, such as IL-10 and transforming growth factor (TGF)-β, and low levels of proinflammatory cytokines, to facilitate tumor evasion (2, 3). The pan-macrophage marker CD68 is now generally utilized to identify tumor-associated macrophages (TAMs) in diagnostic biopsy samples, and CD163 and CD206 are used to identify M2 macrophages (3).

Monocarboxylate transporters (MCTs) are proteins located primarily in the plasma membrane that transport monocarboxylates bidirectionally depending on the concentration gradient of their substrates, including lactate, pyruvate, and ketone bodies (4). MCT1 is ubiquitously expressed in normal tissues, such as gut epithelium (5), heart and red skeletal muscle fibers (6), as well as in various cancer types, including breast cancer (4), melanoma (7), and prostate cancer (8). MCT1 can mediate lactate influx as well as efflux, while MCT4 mainly facilitates the efflux of lactate to maintain steady intracellular pH (9). In high-lactate microenvironment, MCT4 is the major exporting transporter of lactate (10), and high expression of MCT1 on macrophages regulates the lactate uptake and induces M2-like polarization of macrophages (11). LPS and TNFα stimulate the expression of MCT1 in macrophages (12). Macrophages increase the uptake of lactate through MCT1, the possible reason is that lactate can be utilized as energy source to generate ATP to meet the need for production and secretion of cytokines. However, the expression of MCT1 on tumor-associated macrophages is still unknown.

Lactate, generated by glycolytic tumor cells and immune cells, such as macrophages, and dendritic cells (13), is involved in almost all of the main processes following carcinogenesis, including immune evasion, angiogenesis, cell metastasis, and metabolism (14). Functionally, a high lactate concentration serves as an immune suppressor. Lactate derived from tumor cells suppressed the proliferation and cytokine generation of cytotoxic T lymphocytes (CTLs) (15). In addition, the lactate concentration in cancerous tissues was increased almost 10-fold compared to that of healthy tissues. Lactate taken up by macrophages can also induce alternative polarization of macrophages through hypoxia-inducible factor 1α (HIF-1α) stabilization and the resulting increased production of vascular endothelial growth factor (VEGF) (16).

Here, we focus on the expression levels of MCT1 and CD163 on macrophages in breast cancer specimens to investigate the correlation between the expression of MCT1 on macrophages, macrophage phenotypes, and survival outcomes to explore the impact of tumor metabolic reprogramming on the remodeling of the immune microenvironment.

## Materials and Methods

### Tissue Specimens

A total of 108 formalin-fixed paraffin-embedded (FFPE) tissue samples of breast cancer were collected from Renmin Hospital of Wuhan University, People's Republic of China, and 12 cases of benign breast disease were used as controls. Clinical information was extracted from medical records and pathology reports, and the detailed clinicopathological characteristics of the patient are shown in Table 1. Patients were all followed-up for at least 5 years from the date of first diagnosis. All patients involved in the study have written an informed consent form, and this study was approved by the Institutional Ethics Committee of the Renmin Hospital of Wuhan University (approval no. 2018K-C09). Patients did not receive any financial compensation. All methods were performed in accordance with the relevant guidelines and local regulations.

**Table 1:** Clinicopathological associations of MCT1&CD163 expression in breast cancer.

**Clinicopathological parameters**	**CD163 margin**		**CD163 tissue**		**MCT1**		**MCT1—CD163 margin**		**MCT1—CD163 Tissue**	
	**High expression (%)**	***p***	**High expression (%)**	***p***	**High expression (%)**	***p***	**High expression (%)**	***p***	**High expression (%)**	***p***
Age at diagnosis, y		0.333		0.645		0.298		0.350		0.750
≤ 50	33 (50)		10 (58.8)		29 (49.2)		22 (46.8)		6 (50.0)	
≥51	33 (50)		7 (41.2)		30 (50.8)		25 (53.2)		6 (50.0)	
Tumor size (cm)		**0.005**		0.239		0.183		**0.025**		0.322
<2	17 (25.8)		4 (23.5)		18 (30.5)		11 (23.4)		3 (25.0)	
≥2	49 (74.2)		13 (76.5)		41 (69.5)		36 (76.6)		9 (75.0)	
Lymph node metastasis		**0.005**		0.283		0.471		0.062		0.191
Negative	24 (36.4)		6 (35.3)		26 (44.1)		16 (34.0)		3 (25.0)	
Positive	42 (63.6)		11 (64.7)		33 (55.9)		31 (66.0)		9 (75.0)	
Vascular invasion		0.545		0.698		0.091		0.469		0.198
Negative	59 (89.4)		15 (88.2)		51 (86.4)		40 (85.1)		10 (83.3)	
Positive	7 (10.6)		2 (11.8)		8 (13.6)		7 (14.9)		2 (16.7)	
ER		0.069		0.053		0.051		**0.038**		0.054
Negative	37 (56.1)		12 (70.6)		34 (57.6)		29 (61.7)		8 (66.7)	
Positive	29 (43.9)		5 (29.4)		25 (42.4)		18 (38.3)		4 (33.3)	
PR		**0.006**		**0.004**		**0.004**		**0.002**		**0.002**
Negative	40 (60.6)		14 (82.4)		37 (62.7)		32 (68.1)		10 (83.3)	
Positive	26 (39.4)		3 (17.6)		22 (37.3)		15 (31.9)		2 (16.7)	
HER2		0.058		0.238		0.086		0.062		0.196
Negative	46 (69.7)		11 (64.7)		41 (69.5)		31 (66.0)		8 (66.7)	
Positive	20 (30.3)		6 (35.3)		18 (30.5)		16 (34.0)		4 (33.3)	
Molecular subtypes		**0.004**		**0.038**		0.088		**0.007**		**0.001**
Luminal A	10 (16.9)		14 (21.2)		1 (5.9)		23 (48.9)		10 (83.3)	
Luminal B	15 (25.4)		15 (22.7)		4 (23.5)		7 (14.9)		1 (8.3)	
HER2	10 (16.9)		10 (15.2)		4 (23.5)		4 (8.5)		0 (0.0)	
Basal-like	24 (40.7)		27 (40.9)		8 (47.1)		13 (27.7)		1 (8.3)	
Ki67		**0.002**		**0.006**		**0.000**		**0.000**		**0.000**
<14%	24 (36.4)		3 (17.6)		18 (30.5)		13 (27.7)		0 (0.0)	
≥14%	42 (63.6)		14 (82.4)		41 (69.5)		34 (72.3)		12 (100.0)	
Recurrence		**0.012**		0.191		**0.001**		**0.001**		**0.006**
No	38 (57.6)		9 (52.9)		31 (52.5)		23 (48.9)		5 (41.7)	
Yes	28 (42.4)		8 (47.1)		28 (47.5)		24 (51.1)		7 (58.3)	

* *P-values calculated by Log-rank testing; Bold if statistically significant, P < 0.05. ER, estrogen receptor; PR, progesterone receptor; HER2, human epithelial growth factor receptor-2*.

### Immunohistochemistry

A series of 108 paraffin-embedded human breast cancer specimens was characterized by histopathology at Renmin Hospital of Wuhan University from 2011 to 2013. Immunohistochemistry (IHC) staining was performed as follows: deparaffinization, antigen retrieval, blocking (2% bovine serum albumin, 37°C, for 30 min), incubation with the primary antibody (dilution 1:100, 37°C for 2 h), washing, blocking, incubation with the horseradish peroxidase (HRP)-conjugated secondary antibody (dilution 1:500, 37°C for 30 min), washing, and staining with diaminobenzidine (DAB). The specimen incubated without the selective antibody was used as the negative control. And we used the paraffin-embedded human non-small cell lung cancer (CD163) or paraffin-embedded human liver tissue (MCT1) as positive control provided by the antibody companies. The staining results were scored by two independent pathologists based on both the proportion of positively stained tumor cells and the intensity of staining. According to the expression, the protein expression level of CD163 was described according to the numbers of CD163^+^ macrophages using software Image-Pro plus, while the expression level of MCT1 was described according to the percentage of positive cells calculating by the software ImageJ (17, 18). The proportion of tumor cells was scored as follows: 0 (<10% positive cells), 1 (10–20% positive cells), 2 (21–50% positive cells) or 3 (more than 50% positive cells). The intensity of protein expression was determined as follows: 0 (no staining), 1 (weak staining, light brown), 2 (moderate staining, brown), or 3 (strong staining, dark brown). The protein staining positivity was determined using the following formula: overall score = percentage score × intensity score. In addition, the numbers of CD163^+^ macrophages were counted in 10 random fields of each breast cancer specimen at 400× magnification. Receiver operating characteristic (ROC) analysis was used to determine the optimal cut-off values of all protein expression levels in regard to survival rate.

### Immunofluorescence Imaging

Immunofluorescence (IF) imaging was performed to investigate the localization of MCT1 and CD163 as well as the colocalization of CD68 (a marker of all macrophages) and CD163. Tissue specimens undergoing IF staining were incubated with a Cy3-conjugated secondary antibody or a FITC-conjugated secondary antibody for 1 h at room temperature, followed by counterstaining with DAPI for 5 min. Images were captured using a fluorescence microscope (Olympus BX63; Olympus Corporation).

### Analysis of Gene Expression Data

The expression data of breast cancer cases were downloaded from The Cancer Genome Atlas (TCGA) database to analyze the correlation between the mRNA expression of MCT1 (SLC16A1) and CD163 in breast cancer patients. In addition, the association between MCT1 and CD163 mRNA levels and survival outcomes of patients with breast cancer was analyzed.

### Statistical Analysis

Statistical analyses were performed and survival probabilities were determined with SPSS 22.0 (IBM Corporation, Armonk, NY, USA). The relationships between MCT1 and CD163 and the clinical characteristics of patients with breast cancer were evaluated by the Chi-square test. Kaplan-Meier analysis was utilized to calculate the patient survival probability, and the log-rank test was used to assess the heterogeneity in the survival data for each prognostic factor. Multivariate Cox proportional hazard regressions were used to obtain hazard ratios (HRs) and their respective 95% confidence intervals to show the strength of the estimated relative risks. Pearson's correlation analysis was used to evaluate the correlation between MCT1 and CD163 expression levels. Significance levels were set at a *p* < 0.05. All tests were two-sided.

## Results

### Significant Differences Existed in the Expression of CD163 Between the Tumor Invasive Margin and Malignant Tissues

**Figure 1:**
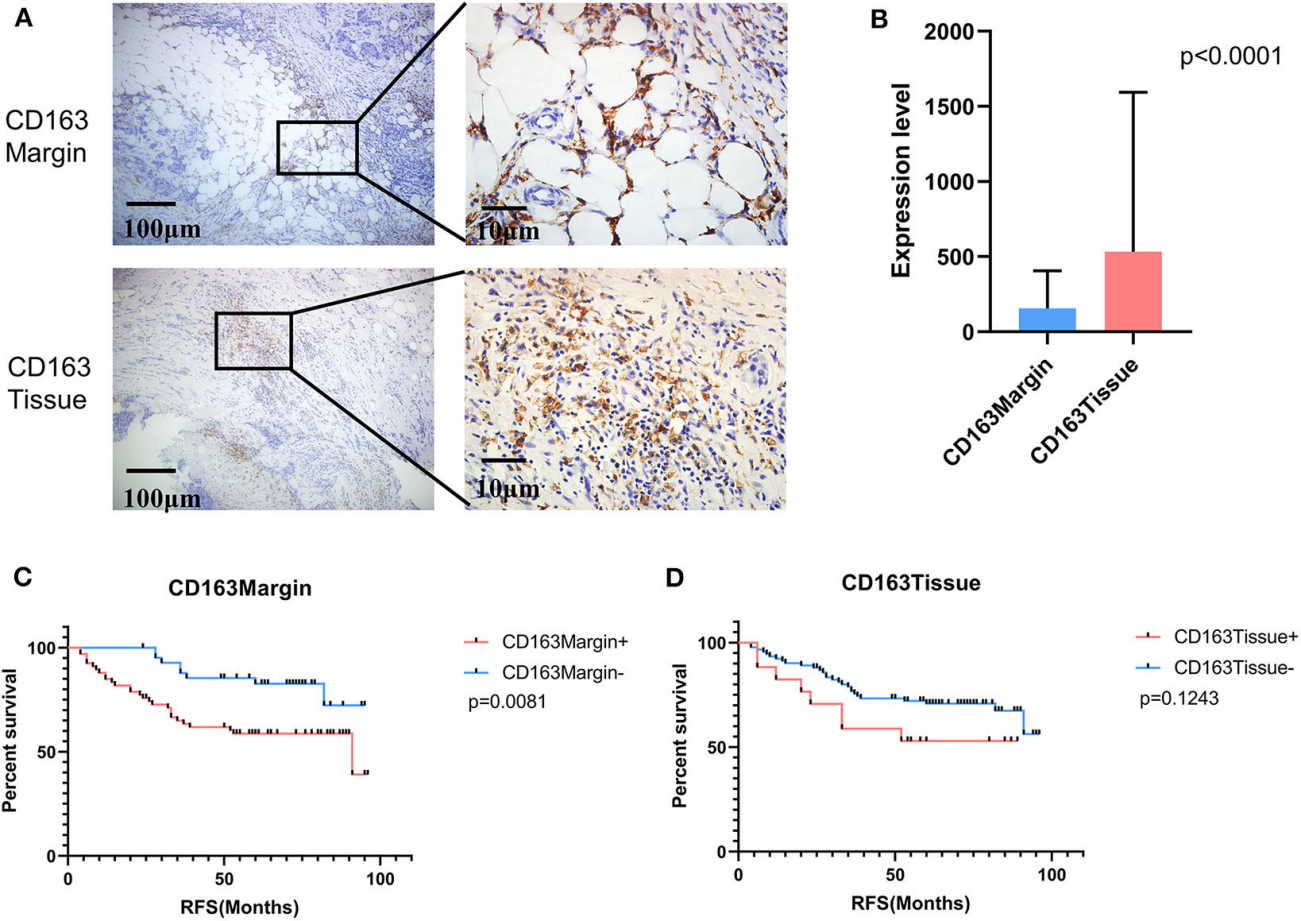
The expression of CD163 in tumor invasive margins and malignant tissues was associated with recurrence-free survival (RFS). **(A)** The positive expression of CD163 in tumor invasive margins and malignant tissues, respectively. **(B)** A comparison of the expression levels of CD163 in tumor invasive margins and malignant tissues. **(C,D)** Kaplan-Meier survival analysis of patients with CD163-positive and -negative IHC staining in the margin or tumor tissues, respectively.

Tumor-associated macrophage (TAM) were phenotypically different between the invasive margin and the core in malignant tumors (19, 20). In colorectal carcinoma, strong infiltration of intraepithelial CD163^+^ macrophages was correlated with unfavorable clinicopathological features, such as lymph node invasion (21); however, in endometrial cancer, stromal TAMs rather than tumor core TAMs promoted lymph node metastasis (22). Therefore, we investigated whether this discrepancy also existed in breast cancer tissues. Immunohistochemistry staining was utilized to examine the expression level of CD163 in 108 cases of primary breast cancer and 12 cases of benign breast disease. As shown in Figure 1A, CD163 protein was positively expressed in both the tumor tissues and the invasive margin near adipose tissues. Of 108 breast cancer specimens, 66 (61.1%) exhibited high expression of CD163 in the margin (CD163Margin), whereas only 2 (16.7%) specimens of benign breast disease showed high expression. However, for the expression of CD163 in the tumor tissues (CD163Tissue), only 17 (15.7%) cases of breast cancer and no (0%) cases of benign breast disease showed high expression. There are significant differences in CD163 protein expression in the margin or tumor tissue between 108 breast cancer specimens and 12 controls (Supplementary Figure 1A, *p* = 0.0015, *p* = 0.0002, respectively). The significant difference also existed between the expression level of CD163 in the margin and that in tumor tissues, and the expression of CD163Tissue was much higher than that of CD163Margin (*p* < 0.0001) (Figure 1B). In addition, Table 1 shows the association between CD163 expression and the clinicopathological features of breast cancer patients. Our results demonstrated that compared with low expression of CD163Margin, high expression of CD163Margin was significantly associated with larger tumor size (*p* = 0.005), lymph node metastasis (*p* = 0.005), PR status (*p* = 0.006), and higher Ki67 (*p* = 0.002), which indicated that CD163Margin might be a predictor of prognosis for breast cancer patients. On the other hand, high expression of CD163Tissue was only significantly related to PR status (*p* = 0.004) and higher Ki67 (*p* = 0.006). No correlations were detected between CD163Tissue and other clinicopathological features, including age, tumor size, lymph node metastasis, vascular invasion, ER status, and human epithelial growth factor receptor-2 (HER2) status. Moreover, Kaplan–Meier analysis and the log-rank test showed that high expression of CD163Margin had a significant association with decreased recurrence-free survival (RFS) (Figure 1C). Multivariate Cox proportional hazard regressions showed that CD163Margin was an independent prognostic predictor in breast cancer (Figure 4, *p* = 0.016; HR = 2.705, 95% CI 1.203–6.083). However, such a relationship was not observed between the expression of CD163Tissue and RFS (Figures 1D, 4).

### CD163 Overexpression Was Found in HER2 Breast Cancer Patients

**Figure 2:**
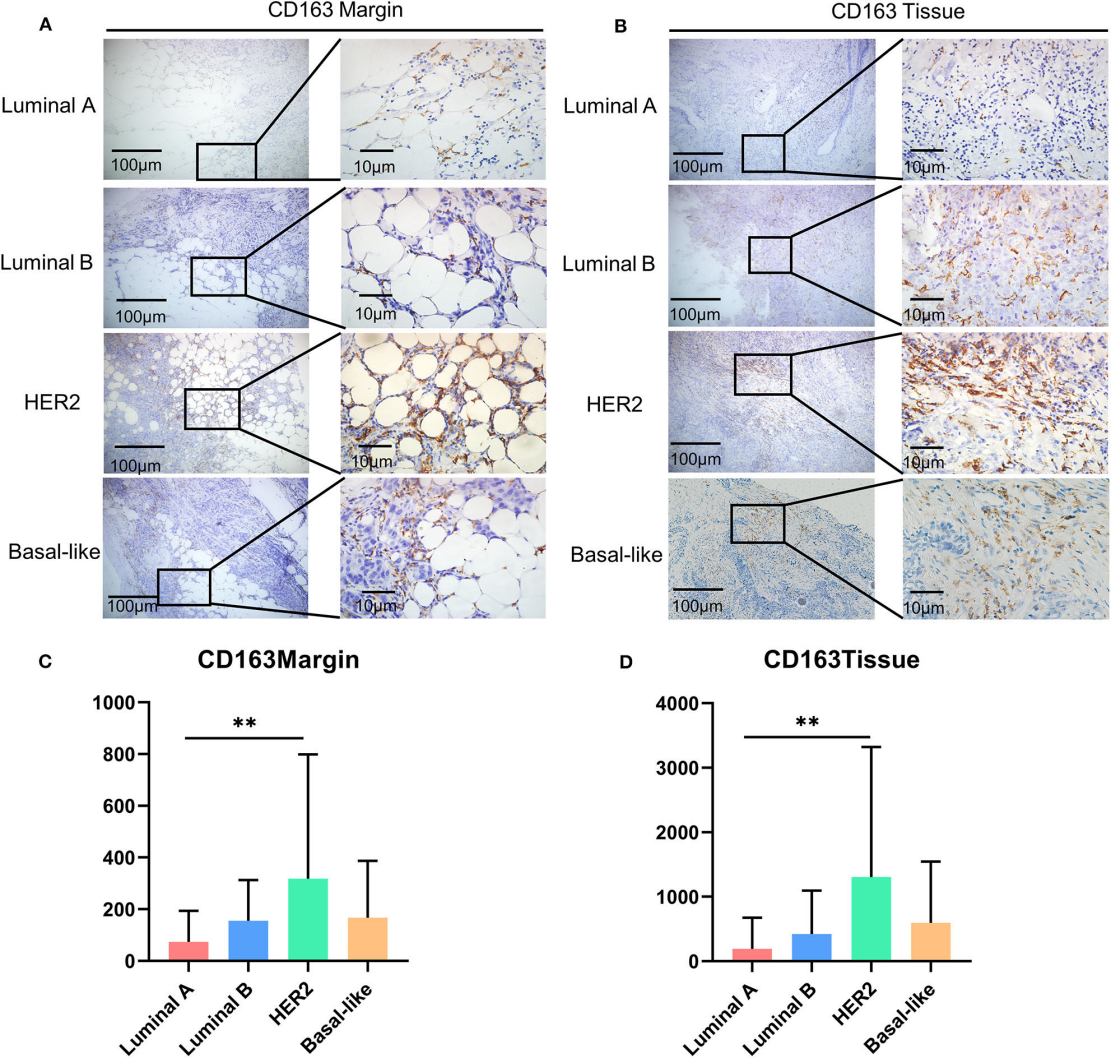
The expression of CD163 among molecular subtypes of breast cancer. **(A)** Representative images of CD163 protein abundance in the margin in different subtypes. **(B)** A comparison of expression levels of CD163 in the margin in different subtypes. **(C)** Representative images of CD163 protein abundance in malignant tissues from different subtypes. **(D)** A comparison of CD163 expression levels in malignant tissues from different subtypes. **p < 0.01.

It has been reported that there are significant differences in the types and numbers of tumor-infiltrating lymphocytes (TILs) among different molecular subtypes of breast cancer (23, 24). Therefore, we compared the infiltration level of CD163^+^ macrophages in different breast cancer subtypes. As for CD163 in the tumor margin, the HER2 subtype had the highest expression level (Figures 2A,B), and a similar condition was observed in the expression level of CD163 in the malignant tissue (Figures 2C,D).

### Increased Infiltration of MCT1^+^CD163^+^ Macrophages Was Associated With Poor Prognosis in Breast Cancer

Previous observations have shown that high expression of MCT1 is significantly correlated with poor prognosis in breast cancer (Supplementary Figure 1D) (25). Here, we further explored the correlation between the expression of MCT1 and CD163 and whether MCT1 has an impact on the polarization of macrophages to promote the expression of CD163. For this purpose, we performed immunohistochemistry (IHC) staining and immunofluorescence (IF) staining to detect the expression of MCT1 and CD163 in a series of 108 cases of breast cancer. We compared the expression of CD163 in MCT1^−^ and MCT1^+^ groups, and the results demonstrated that the margin and tissue expression of CD163 were higher in MCT1^+^ group than that of MCT1^−^ group (Supplementary Figure 1E, *p* = 0.0072, *p* = 0.0166, respectively).

**Figure 3:**
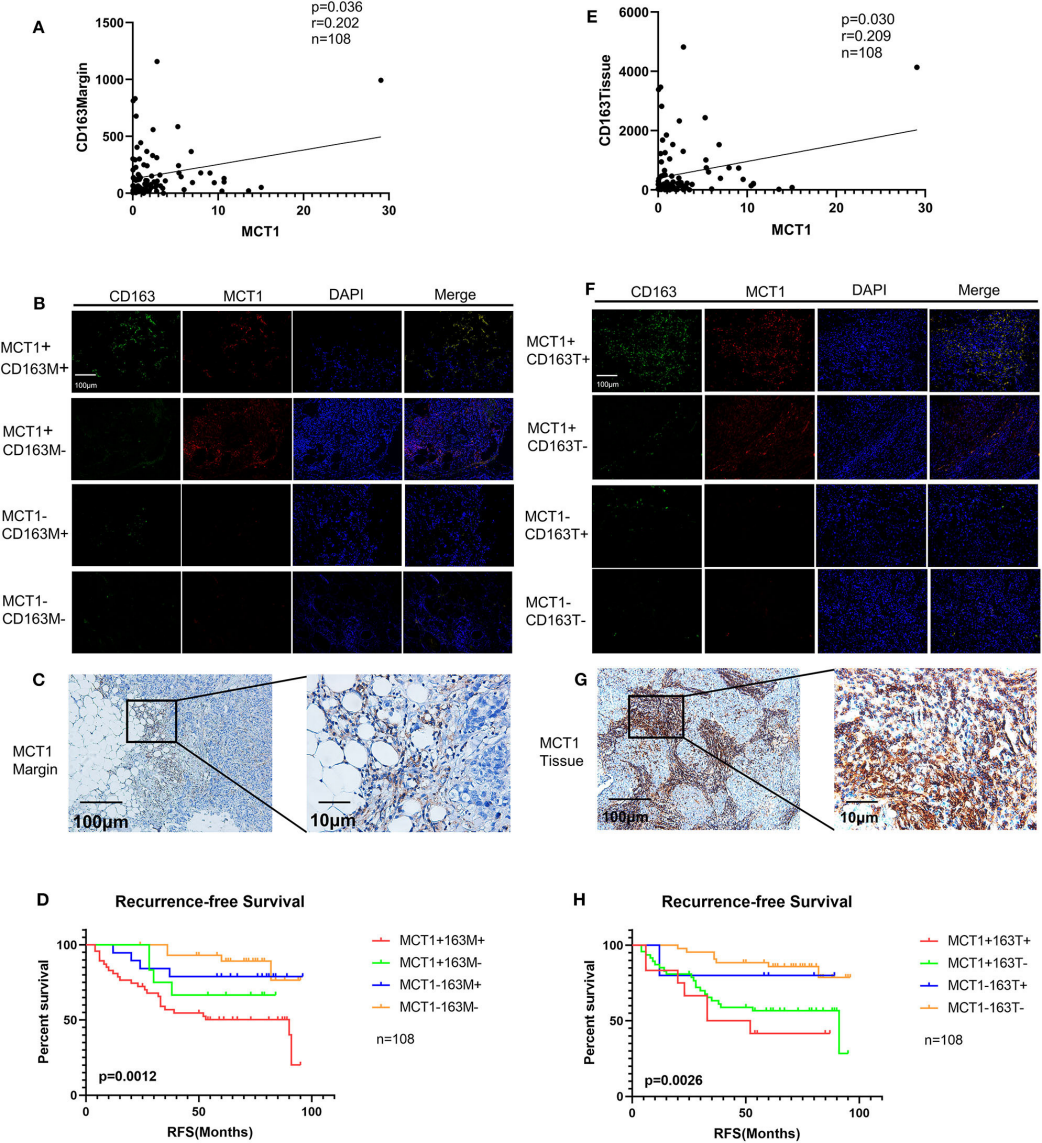
Increased infiltration of MCT1^+^CD163^+^ macrophages is correlated with poor prognosis. **(A)** Correlation analyses between the protein expression levels of MCT1 and CD163 in the margin. **(B)** Representative IF images of MCT1 and CD163 in the margin (red immunofluorescent signal for MCT1 and green immunofluorescent signal for CD163). **(C)** Representative images of MCT1 in the margin. **(D)** Kaplan-Meier survival analysis of patients with biomarker-positive and -negative IHC staining in the margin. **(E)** Correlation analyses between the protein expression levels of MCT1 and CD163 in malignant tissues. (**F)** Representative IF images of MCT1 and CD163 in malignant tissues. **(G)** Representative image of MCT1 in malignant tissues. **(H)** Kaplan-Meier survival analysis of patients with biomarker-positive and -negative IHC staining in the tissues.

**Figure 4:**
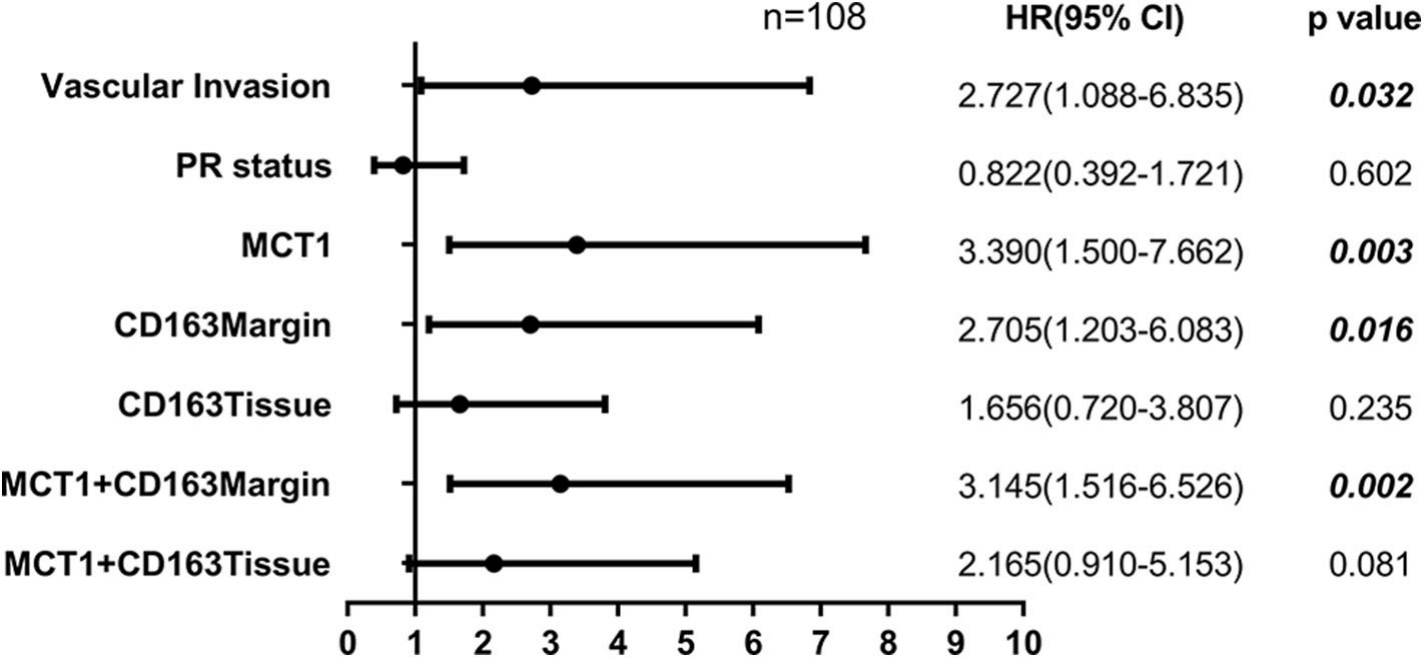
Forest plot showing the results of multivariate Cox proportional hazard regression analyses.

Pearson's correlation analysis revealed that MCT1 expression was positively correlated with the level of CD163Margin (Figure 3A, *r* = 0.202, *p* = 0.036). The IHC and IF results also revealed that MCT1 was frequently present in the margin between tumor tissues and adipose tissues, accompanied by positive expression of CD163 (Figures 3B,C). In addition, IF images showed that the expression of MCT1 was almost completely coincident with that of CD163, which meant that macrophages were both MCT1-positive and CD163-positive (Figure 3B). Overall, 47 (43.5%) breast cancer specimens exhibited high expression of both MCT1 and CD163Margin, and this combined high expression was significantly correlated with tumor size (*p* = 0.025), ER status (*p* = 0.038), PR status (*p* = 0.002), and increased Ki67 staining (*p* < 0.0001) (Table 1). Kaplan-Meier analysis revealed that patients with high infiltration of MCT1^+^CD163^+^ macrophages in the margin displayed shorter RFS than patients with negative expression of both markers or positive expression of one marker (Figure 3D, *p* = 0.0012). Furthermore, multivariate Cox proportional hazard regressions showed that high expression of both MCT1 and CD163Margin was an independent prognostic factor for poor prognosis in breast cancer (Figure 4, *p* = 0.002; HR = 3.145, 95% CI 1.516–6.526; *n* = 108). These observations indicate that high infiltration of MCT1^+^CD163^+^ macrophages in the margin can be a useful biomarker for predicting rapid progression.

MCT1 expression was also positively associated with the level of CD163Tissue (Figure 3E, *r* = 0.209, *p* = 0.030). The IHC and IF results showed similar co-occurrence of MCT1 and CD163 staining in the malignant tissue (Figures 3F,G). However, only 12 (11.1%) specimens had high expression of both MCT1 and CD163Tissue, and this combined expression only displayed a significant association with PR status (*p* = 0.002) and increased Ki67 staining (*p* < 0.0001) (Table 1). The Kaplan-Meier analysis showed similar results: the RFS of patients with high infiltration of MCT1^+^CD163^+^ macrophages in the tissue was much shorter (Figure 3H, *p* = 0.0026) than that of patients with low infiltration of MCT1^+^CD163^+^ macrophages in the tissue. Multivariate Cox proportional hazard regressions indicated that high infiltration of MCT1^+^CD163^+^ macrophages in the tissue might not be an independent predictor of poor RFS (Figure 4, *p* = 0.081; HR = 2.165, 95% CI 0.910–5.153; *n* = 108). These findings suggest that high infiltration of MCT1^+^CD163^+^ macrophages in the tissue is not superior to high infiltration of MCT1^+^CD163^+^ macrophages in the margin for the prediction of breast cancer progression.

### Validation in the TCGA Database

To explore whether the correlation between MCT1 and CD163 also existed in additional breast cancer cases, we downloaded breast cancer expression files from The Cancer Genome Atlas (TCGA) database. Although mRNA expression of MCT1 or CD163 alone was not significantly associated with overall survival (data not shown) or recurrence-free survival (Supplementary Figures 2A,B), there was a significant correlation between MCT1 and CD163 expression (Supplementary Figure 2C) and between the high mRNA expression of both MCT1 and CD163 and shorter overall survival (Supplementary Figure 2D), which may be a potential prognostic marker for breast cancer.

## Discussion

There are increasing studies concentrating on tumor-infiltrating lymphocytes, including T lymphocytes, macrophages and mast cells, as well as the spatial distribution of these cells (21, 22, 26). Tumor-associated macrophages are important cells involved in the tumor microenvironment and participate in tumor progression, immune suppression, metastasis, and tumor angiogenesis through cross-talk with tumor cells and other stromal cells. Here, we showed that the expression of MCT1 and CD163 on macrophages in the infiltration boundary of breast cancer was significantly increased and can be regarded as a useful biomarker for predicting rapid progression. Likewise, overexpression of both MCT1 and CD163 by macrophages in the adjacent tissue may serve as a high-risk factor for poor prognosis in breast cancer patients.

The impacts of TAMs on clinicopathological features and survival outcomes partially depend on their spatial distribution (27, 28), which is consistent with the finding of the present study that high numbers of CD163^+^ macrophages are an unfavorable clinicopathological feature. In gastric cancer, the number of infiltrating macrophages in the malignant tissues was much higher than that in peritumoral tissues; however, infiltration of TAMs into the tumor core was not correlated with any clinicopathological characteristics, but the presence of TAMs in the invasive front was associated with poor prognosis and unfavorable survival (27, 28). The TAMs exhibited a more M2-like phenotype at the margin, while a significant increase in the proportion of M1-like TAMs was observed in the core (20). Mechanically, some studies have speculated that TAMs in the core of tumor are protective because they secrete signals to kill tumor cells (29, 30). In contrast, TAMs located in the invasive margin are immunosuppressive, promote tumor progression and facilitate tumor evasion. Overexpression of cytokines in the TME, such as chemokine (C–X–C motif) ligand 2 (CCL2) and CCL5 (1), contributes to the progression of breast cancer. CCL2 recruits more macrophages into the tumor to promote lymphatic metastasis via VEGF-C secretion (31). In addition, elevated CCL2 induces the secretion of chemokine (C–X–C motif) ligand 12 (CXCL12) in macrophages, which acts on blood vessels to enhance angiogenesis (32). Moreover, increased CCL5 binding to CCR5 activates the protein kinase B/mechanistic target of rapamycin (AKT/mTOR) signaling pathway to promote tumor cell growth and invasion and induces the production of matrix metalloproteinase (MMPs) by macrophages to decrease adhesion and facilitate migration (33). Our results established that the infiltration of TAMs into the tumor margin rather than into the malignant tissues was significantly associated with poor prognosis in breast cancer patients. Further studies are needed to clarify the potential mechanisms by which TAM spatial distribution influences human solid tumors.

The subtype and number of tumor-infiltrating lymphocytes track with tumor heterogeneity. P53 gene mutation is common in multiple tumors, and inactivating mutations of P53 have been associated with reduced immune infiltration (34). Interestingly, induction of P53 resulted in increased expression of colony-stimulating factor 1 (CSF1), CCL2, CXCL1, and IL-15 as well as of the adhesion molecules intercellular adhesion molecule 1 (ICAM1) and vascular cell adhesion molecule 1 (VCAM1), which further recruit natural killer (NK) cells to trigger tumor regression, arguing that oncogenic pathways might also influence immune cell types. Previous studies revealed that CD163^+^ macrophages were positively correlated with lymph node metastasis, hormone receptor negativity, and Ki67 positivity (35–37). The present study validated this relationship between CD163 expression and clinicopathological features by showing that CD163 expression was lower in PR-positive tumors that had a low proliferation level than in highly proliferative PR-negative tumors, and high CD163 expression was associated with poor survival outcome. In addition, this study showed the highest infiltration level of CD163^+^ macrophages in the HER2 subtype. It has been well-established that in response to the Th2 cytokines interleukin-4 (IL-4) and interleukin-13 (IL-13), macrophages undergo alternative activation, gaining abilities to support tumor growth and inhibit antitumor immunity (38). The expression of IL-4 and IL-13 was similarly correlated with hormone receptor status, and IL-4 was increased in samples with an ER-negative status (39, 40). IL-4 is generated by both tumor cells and stromal cells, and IL-4 neutralization resulted in reduced levels of the chemokines CCL2, CCL11, and CXCL5 in the TME (41). Another study demonstrated that high expression of the plasma membrane receptor for IL-13 (IL-13Rα1) was observed in breast cancer patients with HER2 positivity (42). Therefore, increased levels of IL-4 and IL-13 may partially explain the higher infiltration of CD163^+^ macrophages in the HER2 subtype. Given the wide range of changes in chemokine production associated with dysregulation of the HER2 pathway, additional studies will be needed to investigate which immune cell types are affected in patients with distinct types of cancer.

MCT1 functions as a transporter of lactate and has been reported to be generally expressed in various human tumors, including prostate, colon, breast, and lung tumors (43). In line with a previous study (25), the present study demonstrated that high expression of MCT1 was significantly associated with poor prognostic clinicopathological parameters, including PR-negative status and proliferation, as MCT1 was correlated with Ki-67 positivity. Therefore, MCT1 contributes to the aggressive features and is an independent prognostic factor for breast cancer. In addition to participating in tumor metabolism, as the IF results showed, MCT1 and CD163 were colocalized on macrophages, and MCT1 may participate in the lactate uptake into CD163^+^ macrophages in the high-lactate TME. MCT1 played a role in suppressing the phagocytosis of tumor-associated macrophages (44). In glioblastoma, branched-chain ketoacids excreted from tumor cells were taken up by TAMs through MCT1 and were converted to branched-chain amino acids, which attenuated the phagocytosis by TAMs. Furthermore, lactate, another important substrate of MCT1, can induce alternative polarization of macrophages (45). Mechanically, lactate activated the extracellular regulated protein kinase/signal transducer and activator of transcription 3 (ERK/STAT3) signaling pathway to stimulate M2 macrophage polarization to promote proliferation, migration, and angiogenesis in breast cancer, which were abolished with the suppression of ERK/STAT3 signaling (46). On the other hand, lactate activated macrophage G protein-coupled receptor 132 (Gpr132) to promote an alternatively activated macrophage (M2)-like phenotype, which in turn facilitated cancer cell migration and invasion to promote lung metastasis in breast cancer (47). However, MCT4, which facilitates lactate efflux, was highly expressed in the surrounding stromal cells (48). Therefore, in the tumor invasive margin, macrophages with high expression of MCT1 uptake large amounts of lactate, leading them to have immunosuppressive effects in the TME. However, in the core of tumor tissues, due to an insufficient supply of nutrients, tumor cells preferentially consume lactate, which restricts the uptake of lactate by macrophages, thereby resulting in reduced immunosuppressive effects. The present study revealed that there was a significant correlation between MCT1 positivity and CD163 positivity on macrophages; however, the underlying mechanisms are worthy of further investigation.

This is the first attempt to correlate monocarboxylate transporters with macrophages utilizing immunohistochemistry and immunofluorescence imaging methods. We demonstrated that alternations of metabolic-associated proteins are greatly associated with the infiltration and polarization of macrophages in the TME. Increased infiltration of MCT1^+^CD163^+^ macrophages in the margin, rather than in the malignant tissues, was associated with poor prognosis for breast cancer patients and was an independent risk factor for predicting rapid progression of breast cancer. This increased infiltration will be a promising therapeutic target to impede breast cancer progression.

## Data Availability Statement

All datasets generated for this study are included in the article/Supplementary Material.

## Ethics Statement

The studies involving human participants were reviewed and approved by the Institutional Ethics Committee of the Renmin Hospital of Wuhan University (approval no. 2018K-C09). The patients/participants provided their written informed consent to participate in this study.

## Author Contributions

BL, ZX, and SiS are responsible for collecting and collating documents. BL, QY, and ZL performed these experiments. BL and QW are responsible for writing this article, while ShS is responsible for proofreading and submission. All authors contributed to the article and approved the submitted version.

## Conflict of Interest

The authors declare that the research was conducted in the absence of any commercial or financial relationships that could be construed as a potential conflict of interest.

## Supplementary Material

Supplementary Figure 1 **(A)** Comparison of CD163 protein expression data for 108 breast cancer specimens and 12 controls. **(B)** Comparison of CD163Margin and CD163Tissue in 12 benign controls. **(C)** Comparison of the CD163 expression in the margin and tissues without HER2 subtype sample. **(D)** Kaplan-Meier survival analysis of patients with MCT1-positive and MCT1-negative IHC staining. **(E)** Comparison of the CD163 expression in MCT1^−^ and MCT1^+^ groups.

Supplementary Figure 2 **(A)** Kaplan-Meier survival analysis of patients with SLC16A1 (MCT1)-positive and -negative mRNA expression from the TCGA database. **(B)** Kaplan-Meier survival analysis of patients with CD163-positive and -negative mRNA expression from the TCGA database. **(C)** Correlation analyses between the mRNA expression levels of SLC16A1 and CD163. **(D)** Kaplan-Meier survival analysis of patients with biomarker-positive and -negative samples.

Supplementary Table 1 Antibody information.

Supplementary Table 2 Clinicopathological associations of CD163-Margin expression in breast cancer.

Supplementary Table 3 Clinicopathological associations of CD163-Tissue expression in breast cancer.
