# Supplementary figures and images for: Expression of Monocarboxylate Transporter 1 in Immunosuppressive Macrophages Is Associated With the Poor Prognosis in Breast Cancer
Source: Front Oncol. 2020 Oct 16;10:574787. doi: 10.3389/fonc.2020.574787 (PMC7596686; doi:10.3389/fonc.2020.574787)

Figure 3B

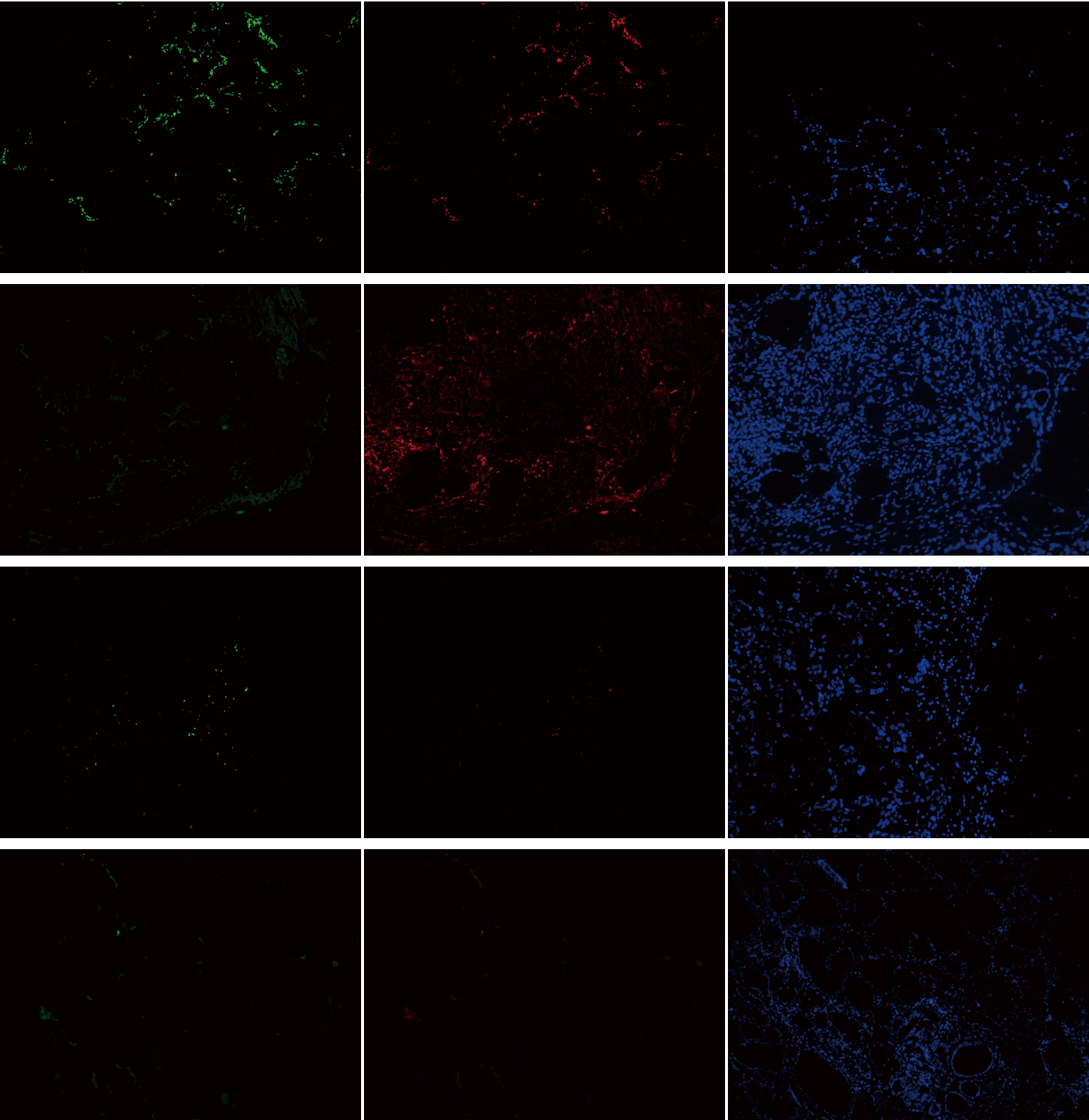

Figure 3F

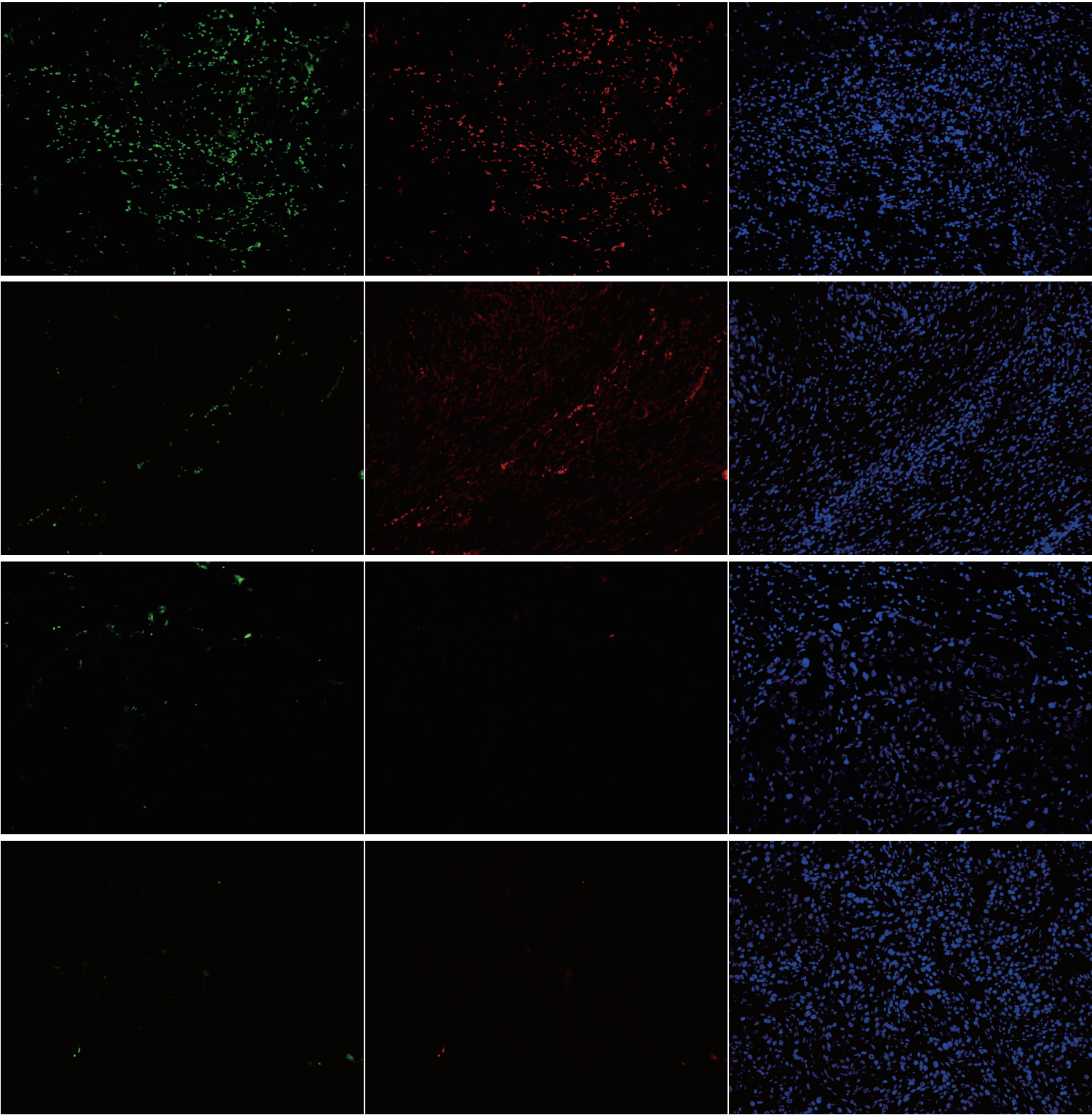

Supplement: Supplementary file 1 [file Data_Sheet_1.PDF]

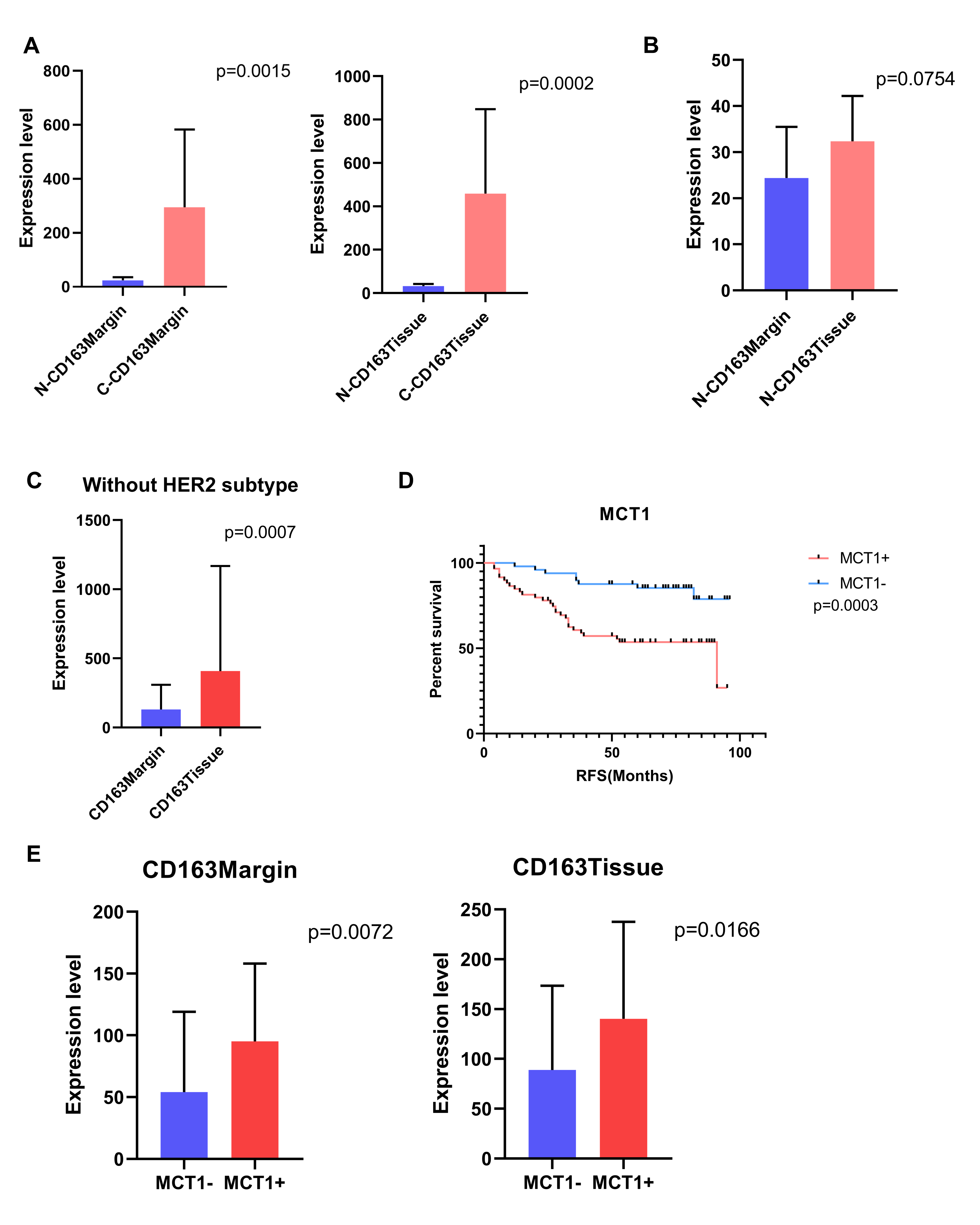

Supplement: Supplementary Figure 1 — (A) Comparison of CD163 protein expression data for 108 breast cancer specimens and 12 controls. (B) Comparison of CD163Margin and CD163Tissue in 12 benign controls. (C) Comparison of the CD163 expression in the margin and tissues without HER2 subtype sample. (D) Kaplan-Meier survival analysis of patients with MCT1-positive and MCT1-negative IHC staining. (E) Comparison of the CD163 expression in MCT1− and MCT1+ groups. [file Image_1.tif]

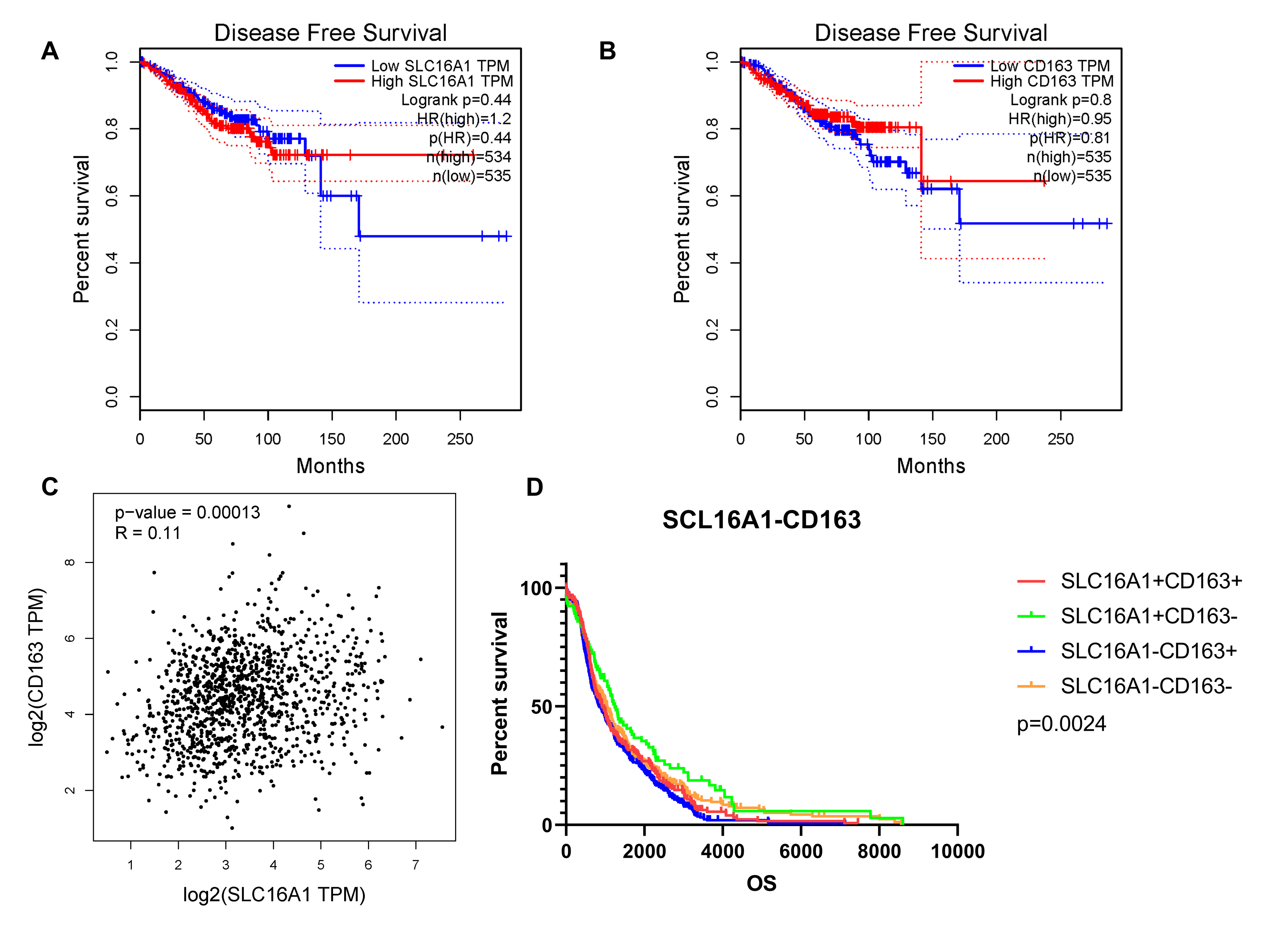

Supplement: Supplementary Figure 2 — (A) Kaplan-Meier survival analysis of patients with SLC16A1 (MCT1)-positive and -negative mRNA expression from the TCGA database. (B) Kaplan-Meier survival analysis of patients with CD163-positive and -negative mRNA expression from the TCGA database. (C) Correlation analyses between the mRNA expression levels of SLC16A1 and CD163. (D) Kaplan-Meier survival analysis of patients with biomarker-positive and -negative samples. [file Image_2.tif]
