# Supplementary material for: Expression of Monocarboxylate Transporter 1 in Immunosuppressive Macrophages Is Associated With the Poor Prognosis in Breast Cancer
Source: Front Oncol. 2020 Oct 16;10:574787. doi: 10.3389/fonc.2020.574787 (PMC7596686; doi:10.3389/fonc.2020.574787)
Supplement: Supplementary Table 1 — Antibody information. [file Table_1.DOCX]

**Table S1. Antibody information**

| Name | Company | NO. |
| --- | --- | --- |
| CD68 | Santa Cruz | sc-70761 |
| CD163 | Cell Signaling Technology | 9332 |
| MCT1 | Santa Cruz | sc-365501 |
