# Supplementary material for: Expression of Monocarboxylate Transporter 1 in Immunosuppressive Macrophages Is Associated With the Poor Prognosis in Breast Cancer
Source: Front Oncol. 2020 Oct 16;10:574787. doi: 10.3389/fonc.2020.574787 (PMC7596686; doi:10.3389/fonc.2020.574787)
Supplement: Supplementary Table 2 — Clinicopathological associations of CD163-Margin expression in breast cancer. [file Table_2.DOCX]

Table S2. Clinicopathological associations of CD163-Margin expression in breast cancer.

| Variables | CD163 Negative | CD163 Positive | P value^*^ |
| --- | --- | --- | --- |
| Survival time (months)  Age at diagnosis, y |  |  | 0.688 |
| ≤50 | 35 (50.0) | 35 (46.7) |  |
| ≥51 | 35 (50.0) | 40 (53.3) |  |
| Tumour siza(cm) |  |  | 0.017 |
| <2 | 34 (48.6) | 22 (29.3) |  |
| ≥2 | 36 (51.4) | 53 (70.7) |  |
| Lymph node metastasis |  |  | 0.000 |
| Negative | 45 (64.3) | 26 (34.7) |  |
| Positive | 25 (35.7) | 49 (65.3) |  |
| Vascular invasion |  |  | 0.121 |
| Negative | 66 (94.3) | 65 (86.7) |  |
| Positive | 4 (5.7) | 10 (13.3) |  |
| ER |  |  | 0.216 |
| Negative | 32 (45.7) | 42 (56.0) |  |
| Positive | 38 (54.3) | 33 (44.0) |  |
| PR |  |  | 0.123 |
| Negative | 34 (48.6) | 46 (61.3) |  |
| Positive  HER2  Negative  Positive  Ki67  <14%  ≥14%  Recurrence  No  Yes | 36 (51.4)  51 (72.9)  19 (27.1)  40 (57.1)  30 (42.9)  54 (77.1)  16 (22.9) | 29 (38.7)  59 (78.7)  16 (21.3)  26 (34.7)  49 (65.3)  42 (56.0)  33 (44.0) | 0.414  0.007  0.007 |
