# Supplementary material for: Expression of Monocarboxylate Transporter 1 in Immunosuppressive Macrophages Is Associated With the Poor Prognosis in Breast Cancer
Source: Front Oncol. 2020 Oct 16;10:574787. doi: 10.3389/fonc.2020.574787 (PMC7596686; doi:10.3389/fonc.2020.574787)
Supplement: Supplementary Table 3 — Clinicopathological associations of CD163-Tissue expression in breast cancer. [file Table_3.DOCX]

Table S3. Clinicopathological associations of CD163-Tissue expression in breast cancer.

| Variables | CD163 Negative | CD163 Positive | P value^*^ |
| --- | --- | --- | --- |
| Survival time (months)  Age at diagnosis, y |  |  | 0.878 |
| ≤50 | 43 (47.8) | 27 (49.1) |  |
| ≥51 | 47 (52.2) | 28 (50.9) |  |
| Tumour siza(cm) |  |  | 0.663 |
| ≤2 | 36 (40.0) | 20 (36.4) |  |
| >2 | 54 (60.0) | 35 (63.6) |  |
| Lymph node metastasis |  |  | 0.091 |
| Negative | 49 (54.4) | 22 (40.0) |  |
| Positive | 41 (45.6) | 33 (60.0) |  |
| Vascular invasion |  |  | 0.448 |
| Negative | 80 (88.9) | 51 (92.7) |  |
| Positive | 10 (11.1) | 4 (7.3) |  |
| ER |  |  | 0.042 |
| Negative | 40 (44.4) | 34 (61.8) |  |
| Positive | 50 (55.6) | 21 (38.2) |  |
| PR |  |  | 0.109 |
| Negative | 45 (50.0) | 35 (63.6) |  |
| Positive  HER2  Negative  Positive  Ki67  <14%  ≥14%  Recurrence  No  Yes | 45 (50.0)  69 (76.7)  21 (23.3)  47 (52.2)  43 (47.8)  63 (70.0)  27 (30.0) | 20 (36.4)  41 (74.5)  14 (25.5)  19 (34.5)  36 (65.5)  33 (60.0)  22 (40.0) | 0.772  0.038  0.217 |
